# Diversifying podiatry placements: The future of podiatry education

**DOI:** 10.1002/jfa2.12006

**Published:** 2024-04-09

**Authors:** Thanaporn Tunprasert, Channine Clarke

**Affiliations:** ^1^ University of Brighton Brighton East Sussex UK

**Keywords:** diverse, education, placements, practice learning, role‐emerging

## 1 BACKGROUND

Podiatry is an allied health profession focusing on the assessment, diagnosis, treatment and prevention of foot, ankle and lower limb conditions. In the United Kingdom context, the recent National Health Service's (NHS) Long Term Workforce Plan has highlighted an ongoing shortfall in the podiatry workforce and the need for increased recruitment to podiatry training [1]. Current available routes to podiatry education in the UK include BSc (Hons) Podiatry, BSc Podiatry Apprenticeship and MSc Podiatry (Pre‐Registration). The curriculum typically covers theoretical knowledge and practical skills relevant to lower limb conditions as per the Health and Care Professions Council's Standards of Proficiency for Chiropodists and Podiatrists [2] and the Royal College of Podiatry's (RCPod) Core Curriculum. In addition, learners are normally required to complete at least 1000 placement hours as part of their degree study. Practice placements are a crucial component of podiatry education. During placements, learners are able to apply their theoretical knowledge to real‐world scenarios with exposure to a range of patient cases. Traditionally, these placements have been concentrated in podiatry clinics and hospitals in the NHS. Due to the ongoing podiatry workforce shortage in the UK [3], there has been an impact on “traditional” clinical placements where fewer placements have been available for podiatry learners. This issue poses a challenge to the sustainability of podiatry education and training pipeline.

## 2 DIVERSE PLACEMENTS

“Diverse placements” is a broad term that can be used to encompass a range of practice learning opportunities in specialized or non‐podiatry settings. These types of placements are widely incorporated within other allied health professions, with literature highlighting key benefits within occupational therapy [4, 5, 6] and physiotherapy [7, 8, 9]. These benefits include the strengthening of professional identity, leadership, communication skills, professional reasoning, evidence‐based practice, and autonomous working. However, there is a lack of evidence on this in the podiatry profession. This innovative approach in placement education is also recognized by the RCPod in its publication on the practice‐based learning framework for pre‐registration podiatry learners [10]. With Advanced Clinical Practitioner and First Contact Practitioner roles centered around the four pillars of practice [11, 12], it is essential to ensure that graduates have opportunities for placement experiences that enable them to develop their research, leadership and education aspects of practice in addition to clinical knowledge and skills. The diverse placement experiences are thus critical to better prepare learners for the diverse roles that are increasingly available to them [10]. It is worth noting that the long‐term effects of diverse placements are yet to be understood, particularly for the podiatry profession, due to its recent development.

## 3 REMOTE PLACEMENTS

Remote placements have proven to be an effective solution to the challenges in healthcare education, particularly during the COVID‐19 pandemic [13]. Examples of remote placements in podiatry include telehealth consultations, triaging activities, simulated case studies and service improvement project work. These placements have shown to improve learners' clinical competence and communication skills, enable more person‐centered approach and foster innovative mindsets [14, 15]. Learners that have undertaken remote placements often take part in the placements from their home through digital platforms appropriate for their placements. This allows for more flexibility (e.g., for those with caring responsibility), reduces placement‐related costs (e.g., travel and accommodation) and broadens access to placements without geographic constraints. With evidence suggesting that financial challenges are the top factor affecting learners' ability to continue with their healthcare training and education [16], remote placements can help to reduce this burden. Hands‐on experiences remain invaluable for podiatrist‐in‐training, but remote placements can offer a complementary option to traditional in‐person clinical placements to enhance the overall placement experiences by providing its own unique perspectives and benefits.

## 4 THE UNIVERSITY OF BRIGHTON MODEL

At the University of Brighton, diverse placements can account for up to 28% of the total placement hours for podiatry learners. This equates to approximately 1–2 blocks of 4 full time weeks of placements. As part of the Brighton model, learners can indicate (1) whether they wish to do diverse placements as part of their study, and (2) preferences for the type(s) of diverse placements they wish to undertake, allowing for optionality in their podiatry degree education. It is important to note that diverse placements can still be clinical placements, but they typically take place in more diverse settings as demonstrated in Table 1.

**TABLE 1:** Examples of diverse placements for podiatry courses at University of Brighton that are already in place or under development.

Diverse placements^a^	
Specialist placements	Role‐emerging placements
Sports podiatry Podiatric surgery Private practices Research Education Leadership	Falls prevention services Homeless services Emergency services Primary care Schools
• International placements	

^a^ The different types of diverse placements can also be found as suggestions in National Health Service [1], Durrant [10] and Royal College of Podiatry [20, 21, 22].

The introduction of diverse placements provides exciting opportunities for learners to explore adjacent aspects of podiatry as a profession beyond a typical podiatry clinical setting. As learners can indicate their preferences for diverse placement options, they are able to tailor their diverse placement experiences to suit their interests and potential future career progression. For example, learners who are interested in becoming a podiatric surgeon can choose a podiatric surgery placement as their preferred option of diverse placements. From the placement providers' perspectives, these placements also help to reduce the pressure on the traditional clinical placements due to staff shortages [3] and offer opportunities for traditional placement providers to offer alternative types of placements which may be of benefit to their services. For example, a traditional placement provider may choose to also offer a leadership placement for learners to work on an audit or service evaluation project.

The diverse placements can also be delivered for up to 100% remotely. For example, a research placement may be delivered 80% remotely for activities related to research project preparation and 20% in‐person for data collection. This is a representation of hybrid working patterns, which provides benefits to workers' well‐being and may help to minimize burnout [17]. An agreed remote activity content is normally required between the placement provider, the learner(s) and the university's podiatry placement team prior to the start of a placement. For role‐emerging placements which take place in non‐podiatry settings, long‐arm supervision [18] is provided by the podiatry placement team. The long‐arm supervisor is a named person who provides additional support (e.g., weekly meetings) but is not with students on a day‐to‐day basis during the placement. This is to ensure inputs from podiatry perspectives, including supporting learners with integration of theory and practice, ensuring adherence to professional standards and codes of conduct and providing professional support and guidance specific to podiatry. Thus, the incorporation of diverse placements into the placement model offers a more balanced and sustainable approach to practice‐based podiatry education [19].

## 5 CONCLUSIONS

In the changing world of podiatry education, diverse placements can provide innovative solutions to several pressing challenges. One of the key benefits is to reduce the pressure of traditional clinical placement demands on the overextended healthcare providers, while allowing for new avenues of specialized learning that can be incorporated into learners' future career progression plans. For example, in podiatric surgery, leadership, research, education and role‐emerging areas. An alternative mode of placement delivery (e.g., remote and hybrid) can also provide several benefits including improving work–life balance, reducing placement‐related costs and accessibility of placements beyond geographical constraints. The University of Brighton model serves as an example of how diverse placements can be integrated into BSc (Hons) Podiatry, BSc Podiatry Apprenticeship and MSc Podiatry (Pre‐Registration) courses. The inclusion of diverse placements in podiatry training and education is vital to prepare a new generation of podiatrists who are more versatile and equipped to handle complex challenges of the future healthcare environment. There is also an opportunity for future research due to the current lack in empirical evidence on podiatry placements and recent development of innovative placement approaches (e.g., non‐clinical placements and online placements) in response to the changing landscape of healthcare education.

## AUTHOR CONTRIBUTIONS

Thanaporn Tunprasert and Channine Clarke prepared, reviewed, edited and approved the manuscript.

## CONFLICT OF INTEREST STATEMENT

None of the authors have any conflicts or competing interests to declare.

## ETHICS STATEMENT

Not applicable.

## CONSENT FOR PUBLICATION

Not applicable.

## Data Availability

Data sharing is not applicable to this article as no new data were created or analyzed in this study.
